# The transcriptome analysis of *Protaetia brevitarsis Lewis* larvae

**DOI:** 10.1371/journal.pone.0214001

**Published:** 2019-03-21

**Authors:** Zhongjie Li, Miaomiao Meng, Shasha Li, Bo Deng

**Affiliations:** Medical College, Henan University of Science and Technology, Luoyang, PR China; Centre de Recherche en Cancerologie de Lyon, FRANCE

## Abstract

Larvae of the pest *Protaetia brevitarsis* are used to treat infections in traditional Chinese medicine. However, genomic information about this non-model species is currently lacking. To better understand the fundamental biology of this non-model species, its transcriptome was obtained using next generation sequencing and then analyzed. A total of 7.62 Gb of clean reads were obtained, which were assembled into 169,087 transcripts corresponding to 142,000 annotated unigenes. These unigenes were functionally classified according to Gene Ontology (GO), euKaryotic Ortholog Groups of proteins (KOG), and Kyoto Encyclopedia of Genes and Genomes (KEGG) annotations. A total of 41,921 unigenes were assigned to 56 GO terms, 21,454 unigenes were divided among 26 KOG categories, and 16,368 unigenes were assigned to 32 KEGG pathways. In addition, 19,144 simple sequence repeats (SSRs) were identified. Furthermore, several kinds of natural antimicrobial peptides and proteins, 4 histones with potential antimicrobial activity, and 41 potential antimicrobial peptide sequences were identified. These data are the first reported whole transcriptome sequence of *P*. *brevitarsis* larvae, which represents a valuable genomic resource for studying this species, thus promoting the utilization of its medical potential.

## Introduction

The beetle species *Protaetia brevitarsis* Lewis is widely distributed in China, and it is a pest of plants, including vegetable crops. In traditional Chinese medicine, its larvae are used to treat microbial infections. However, very little research has been conducted on this species. Moreover, there is also no genomic data from this non-model organism.

Transcriptome data represent an essential type of genome data and offer an opportunity to explore the genomes of non-model organisms. Owing to the rapid development of next generation sequencing (NGS) technologies, such as the Illumina sequencing platform, the transcriptomes of non-model organisms can be characterized quickly, inexpensively, and accurately [1, 2]. Accordingly, the transcriptomes of many non-model organisms have been characterized by NGS, such as the green odorous frog *Odorrana margaretae* [3], the inland robust scorpion *Urodacus yaschenkoi* [4], the land snail *Koreanohadra kurodana* [2], the Chinese red-headed centipede *Scolopendra subspinipes mutilans* [5], and several luminescent beetle species [6].

The *P*. *brevitarsis* larvae live on the ground and feed on rotten food. This type of habitat and diet lead them to encounter many different kinds of pathogenic microbes, which has imposed strong selective pressures on their immune systems. The insect immune system is composed of two elements: cellular defense responses and humoral defense responses [7]. Insect antimicrobial components secreted by the insect humoral immune system, especially antimicrobial peptides and proteins (AMPs), play an important role in defense against microorganisms, which may be the molecular basis of the anti-infection effects of *P*. *brevitarsis* larvae used in medicine. Many insect AMPs have been identified, characterized, and categorized according to familiar sequences and structures, including linear-form, disulfide bridges, and proline- or glycine-rich regions [8]. Additionally, AMPs from insects also show great potential as anti-infection agents [9].

In this study, the transcriptome profile of *P*. *brevitarsis* larvae was constructed using the Illumina sequencing platform. After de novo transcriptome assembly, bioinformatic analyses were conducted to annotate the functional direction of the assembled sequences. The analyses focused on *P*. *brevitarsis* larvae AMPs. This research provides the first large-scale transcriptome data from *P*. *brevitarsis* larvae, which improves the current understanding and medical utilization of this species while enabling the discovery of important functional genes.

## Materials and methods

### Ethics statement

All animal collection and utility protocols were approved by the Henan University of Science and Technology of Biology Animal Use Ethics Committee.

### Insect collection

The *Protaetia brevitarsis* Lewis larvae used in this study were obtained from a breeder in Henan, China. After being washed in 0.9% saline, the samples were immediately frozen in liquid nitrogen and stored at -80°C until use.

### cDNA library construction, Illumina sequencing, de novo assemble, and gene annotation

Total RNA was extracted using TRIzol reagent (Life Technologies, Carlsbad, CA, USA), and cDNA library construction and Illumina sequencing of the samples using an Illumina HiSeqTM 2000 sequencer (Illumina, San Diego, CA, USA) were performed by Novogene Bioinformatics Technology Co., Ltd. (Beijing, China).

De novo transcriptome assembly was conducted using the program Trinity [1]. Gene annotation was based on searches of various public databases, including NCBI Nr (non-redundant protein sequences), NCBI Nt (non-redundant nucleotide sequences), Pfam (Protein family), KOG (euKaryotic Ortholog Groups of proteins), Swiss-Prot (a manually annotated and reviewed protein sequence database), KO (Kyoto Encyclopedia of Genes and Genomes Ortholog database), and GO (Gene Ontology). SSR markers were isolated from the transcriptome using MISA (http://pgrc.ipk-gatersleben.de/misa/misa.html).

A detailed description of cDNA library construction, Illumina sequencing, the de novo assemble and gene annotation is given in S1 File.

### Potential AMP sequences identification

To identify more potential AMPs and peptide sequences with potential antimicrobial activity in the transcriptome of *P*. *brevitarsis* larvae, the assembled unigenes were also BLASTed with known AMPs from The Antimicrobial Peptide Database (APD) [10], Collection of Anti-Microbial Peptides (CAMP) [11], and the Linking Antimicrobial Peptides (LAMP) database [12] using BLAST 2.2.31+ with a sequence similarity cutoff of 80%.

## Results and discussion

### Transcriptome sequencing and de novo assembly

The *P*. *brevitarsis* larvae cDNA library was sequenced using the Illumina HiSeqTM 2000 sequencer, ultimately obtaining 50,796,336 clean reads with 98.09% Q20 and 42.88% GC content after the removal of adaptor sequences, ambiguous nucleotides, and low-quality sequences. The clean reads were assembled into 169,087 transcripts, which corresponded to 142,000 unigenes. A summary of sequencing and assembly results was presented in Tables A and B in S1 File, and the unigene sequences was presented in S2 File. The clean data had been deposited into the Sequence Read Archive (SRA) database of the National Center for Biotechnology Information (NCBI) under the accession number PRJNA516097.

### Functional annotation of unigenes

After public databases were used for sequence similarity searches, 56,937 unigenes (40.09% of all unigenes) were successfully matched to annotations in at least one database (Table C in S1 File), which is similar to rates reported in other beetle species [13–15], indicating that a large number of species-specific genes, noncoding gene regions, and non-conserved domains could not be matched to annotated genes or that chimeric sequences had occurred as a result of assembly errors [16]. Among the annotated unigenes, 25.87% (36,743) had significant matches in the Nr database, 9.07% (12,881) in the Nt database, 11.52% (16,368) in the KO database, 20.23% (28,727) in the Swiss-Prot database, 28.52% (40,511) in the PFAM database, 29.52% (41,921) in the GO database, and 15.1% (21,454) in the KOG database. Furthermore, there were 4,618 unigenes (3.25%) annotated in all seven databases.

**Fig 1:**
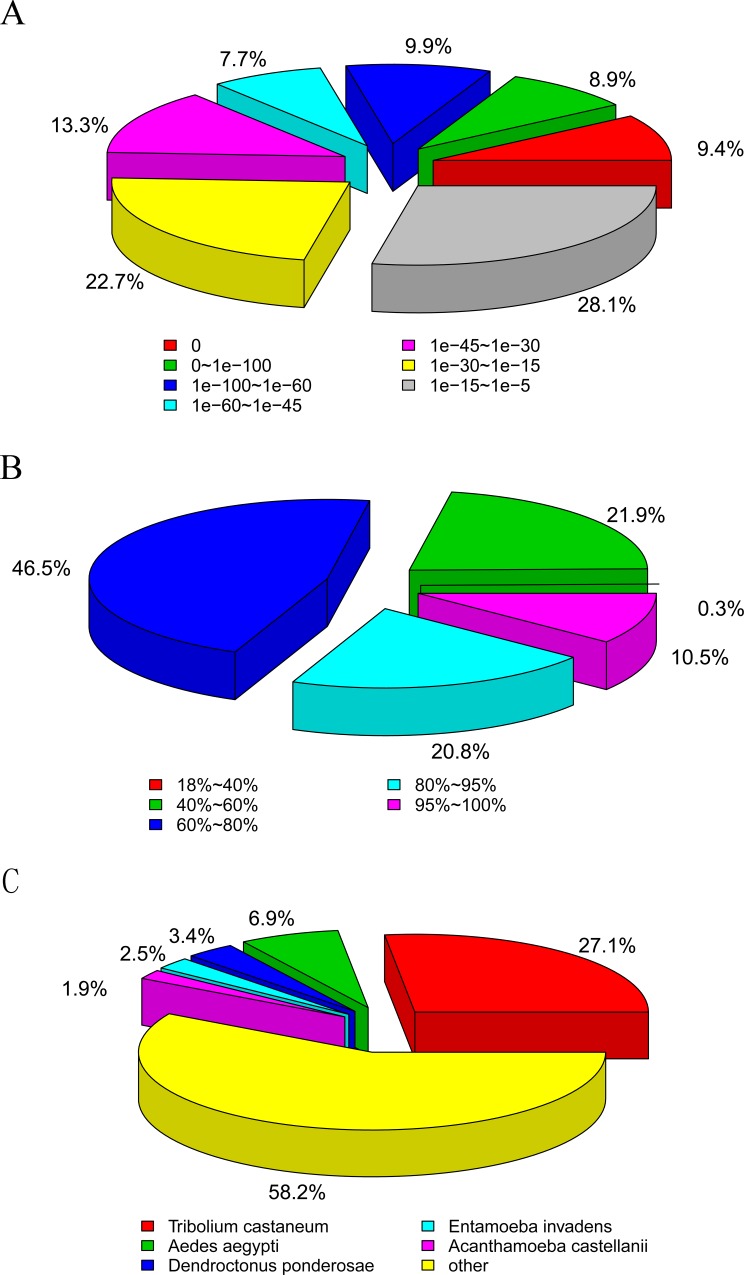
E-value distribution, similarity distribution, and top-hit species of Protaetia brevitarsis Lewis Larvae unigenes blast against Nr database. A: E-value distribution; B: Similarity distribution; C: Top-hit species.

The E-value distribution and similarity distribution of the annotated unigenes in the Nr database identified 49.2% (Fig 1A) of annotated sequences as having strong homology (E-value less than 1E-30), with 77.8% (Fig 1B) of the annotated sequences having a similarity index higher than 60%. The top-hit species distribution showed that 27.1% (Fig 1C) of *P*. *brevitarsis* larvae unigene sequences matched with sequences from red flour beetle *Tribolium castaneum*. This overlap of unigenes was lower than that for other beetle species, such as the pine shoot beetle *Tomicus yunnanensis* (62.48%), the salt marsh beetle *Pogonus chalceus*, and the nipa palm hispid *Octodonta nipae* (72.6%) [17–19]. However, this value was similar with respect to the seven-spot ladybird beetle *Coccinella septempunctata* (28.5%) [15]. In the NCBI protein database, the number of protein sequences from *T*. *castaneum* and the mountain pine beetle *Dendroctonus ponderosae* were 42,319 and 49,666, respectively. But, only 3.4% of *P*. *brevitarsis* larvae unigene sequences matched with those of *D*. *ponderosae*. It was indicated that *Protaetia brevitarsis Lewis* had near evolution distance with *T*. *castaneum*.

**Fig 2:**
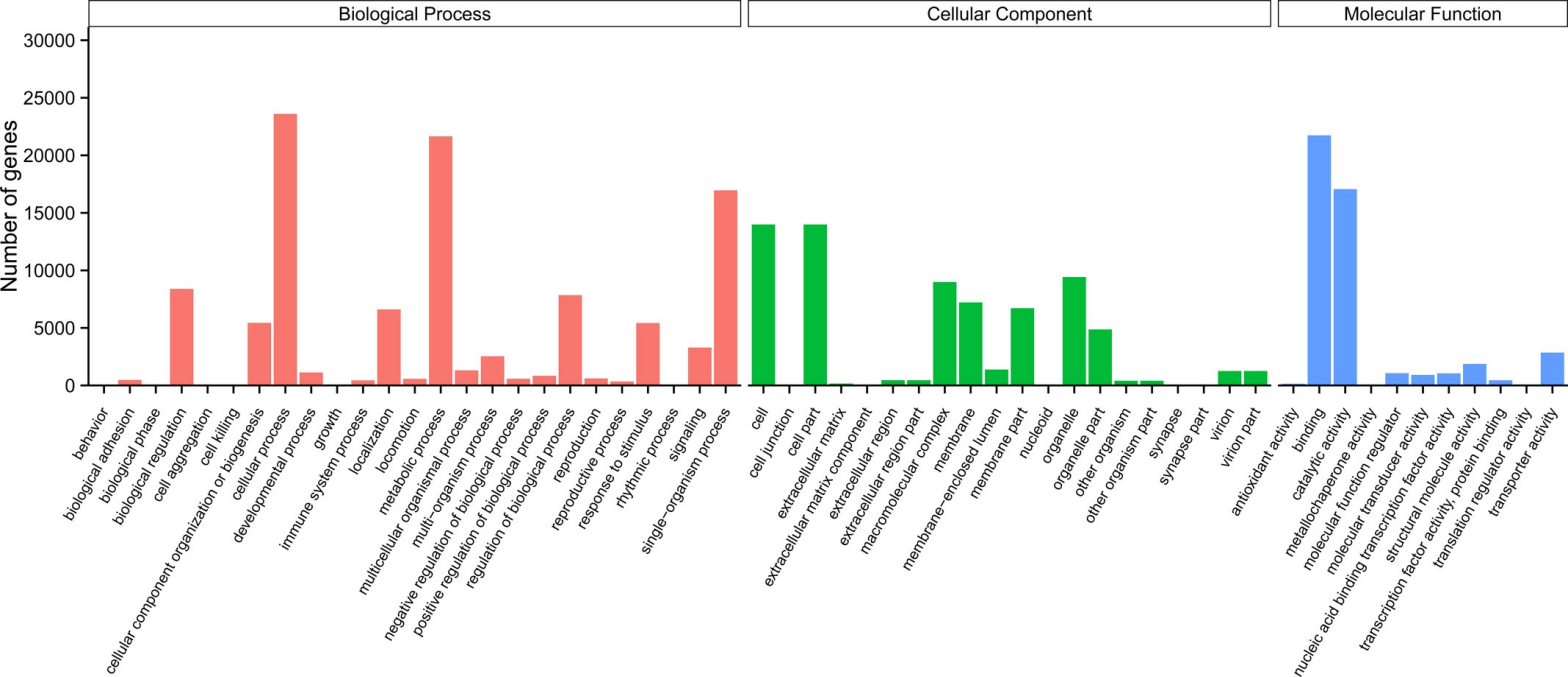
Histogram of the gene ontology (GO) classification of the *Protaetia brevitarsis Lewis* Larvae unigenes. Each annotated sequence could be assigned to more than one GO term.

**Fig 3:**
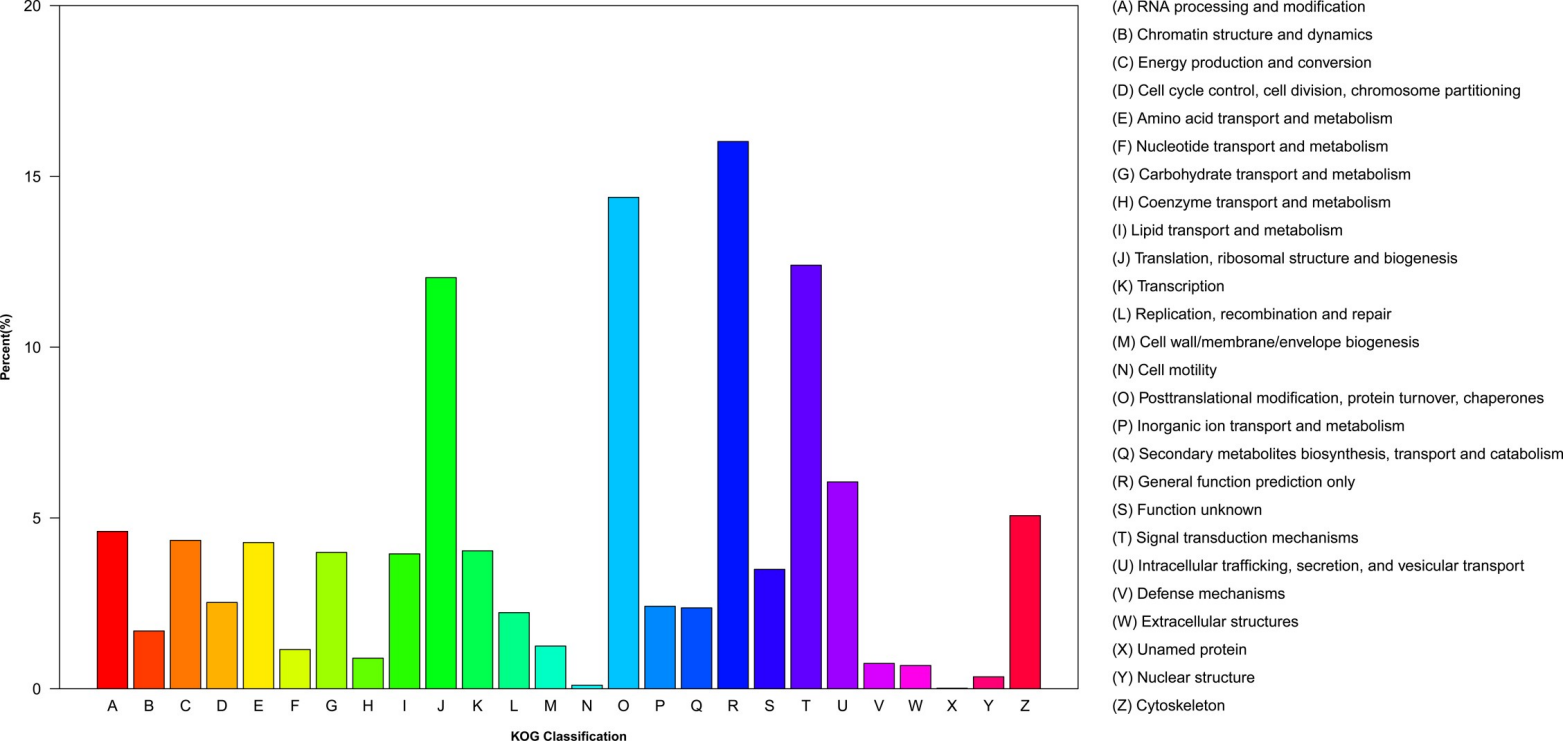
Histogram of the KOG (euKaryotic Ortholog Groups of proteins) classification of the *Protaetia brevitarsis Lewis* Larvae unigenes.

**Fig 4:**
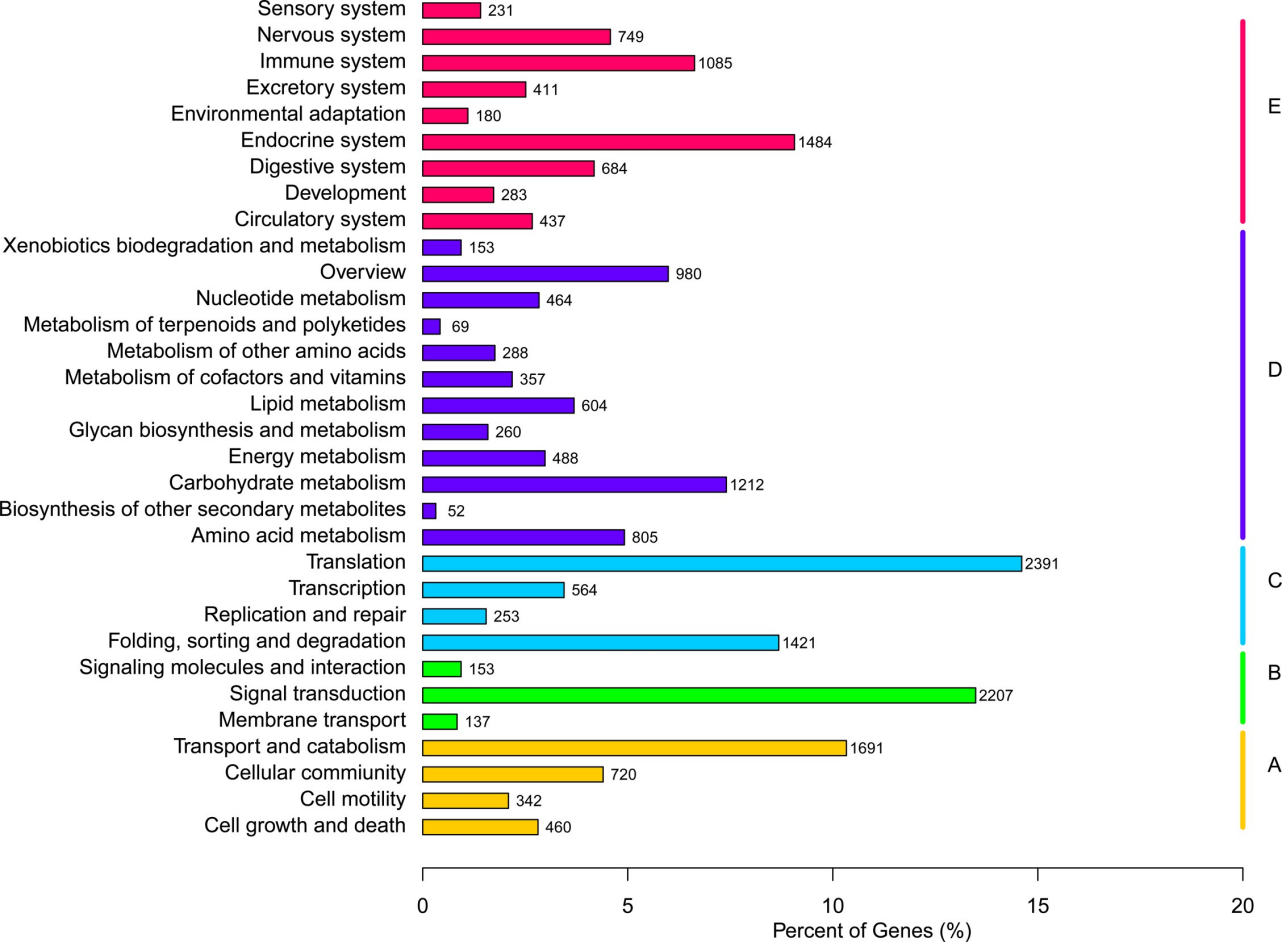
Histogram of the KEGG Pathway classification of the the *Protaetia brevitarsis Lewis* Larvae unigenes. A: Cellular process, B: Environmental information processing, C: Genetic information processing, D: Metabolism, E: Organismal system.

GO categories were used to classify the functions of the unigenes, and the classification of GO categories was conducted using the GOseq R package based on the Wallenius non-central hyper-geometric distribution [20]. According to GO annotation, 41,921 unigenes were classified into three main GO categories: biological process, cellular component, and molecular function (Fig 2). Among the biological process terms, 25 level-2 categories were identified, and cellular process and metabolic process were the most abundant terms. For cellular components, 20 level-2 categories were identified, and cell, cell part, and organelle were the most abundant groups. For molecular function, 11 level-2 categories were identified, and the genes that were associated with binding and catalytic activities were the most represented. Similar gene GO classification distributions had also been reported in transcriptomic studies of other beetles [17, 21, 22]. To further predict putative protein functions, a KOG analysis was performed. Based on our results, 21,454 unigenes were divided into 26 categories (Fig 3), and the largest category was ‘general functional prediction only’ (16.02%), followed by ‘posttranslational modification, protein turnover, chaperones’ (14.38%), ‘signal transduction mechanism’ (12.40%), and ‘translation, ribosomal structure and biogenesis’ (12.04%). The KEGG [23] database was used to identify potential biological pathways represented in the *P*. *brevitarsis* larvae transcriptome, and the classification of KEGG pathways was performed using KOBAS software [24]. A total of 16,368 unigenes were classified into five categories, including cellular processes, environmental information processing, genetic information processing, metabolism, and organismal systems, and then assigned to 32 sub-terms (Fig 4). Among the pathways, translation (2391 unigenes) and signal transduction (2207 unigenes) were most highly represented. Similar gene KEGG classifications distributions had also been found in coconut leaf beetle *Brontispa longissima* [25]. These annotations provide a valuable resource for future research on specific processes, functions, and pathways in *P*. *brevitarsis* and related species.

### SSR detection from the assembled unigenes

As SSR markers are highly polymorphic, co-dominant in their inheritance, highly specific in their amplification, and repeatable in their genotyping, they are widely used in genetic studies to analyze genetic diversity and biological evolution [26]. And transcriptome sequencing using NGS technologies has greatly accelerated the process of discovering molecular markers in non-model organisms [27].

In this study, a minimum of ten repeat numbers for mononucleotide repeats was selected, six for dinucleotide repeats, and five for tri-, tetra-, penta-, and hexa-nucleotide repeats. Thus, 19,144 SSRs were isolated from 13,933 sequences, with 3,136 sequences containing more than one SSR. Additionally, 1,522 SSRs were found in the compound form. Among the identified SSRs, mononucleotide repeats accounted for the highest percentage (13,550 SSRs, 70.77%) followed by trinucleotide repeats (3888, 20.30%), dinucleotide repeats (1575, 8.24%), and tetranucleotide repeats (110, 0.62%). A similar distribution of repeat units were also found in the nipa palm hispid beetle *Octodonta nipae* [28]. Frequency of SSRs based on the number of repeat units in *Protaetia brevitarsis Lewis* Larvae was provided in Table D in S1 File. The five most frequent motif types in the transcriptome were A/T (13,195 SSRs, 68.92%), AAT/ATT (1207, 6.30%), AGG/CCT (1001, 5.23%), AT/AT (861, 4.50%), and AAG/CTT (669, 3.49%). These SSR markers will be useful for assessing genetic diversity and population structure in *P*. *brevitarsis* and related species.

### AMPs

Drug-resistant bacterial pathogens have caused significant health problem worldwide [29, 30]. AMPs have become a particularly promising class of candidates for overcoming this problem, as they have unique modes of action that differ from those of classical antibiotics and are difficult for bacteria to develop resistance to [31, 32].

Natural AMPs play an important role in immune systems, and they protect multicellular organisms by controlling and/or combating pathogens by killing microbes directly or through functions related to diverse immunomodulatory activities [33]. A greatest diversity of AMPs have been identified in different kinds of insects, including beetles [34, 35]. In this study, several kinds of natural AMPs were identified in the *P*. *brevitarsis* larval transcriptome.

#### Lysozymes

**Fig 5:**
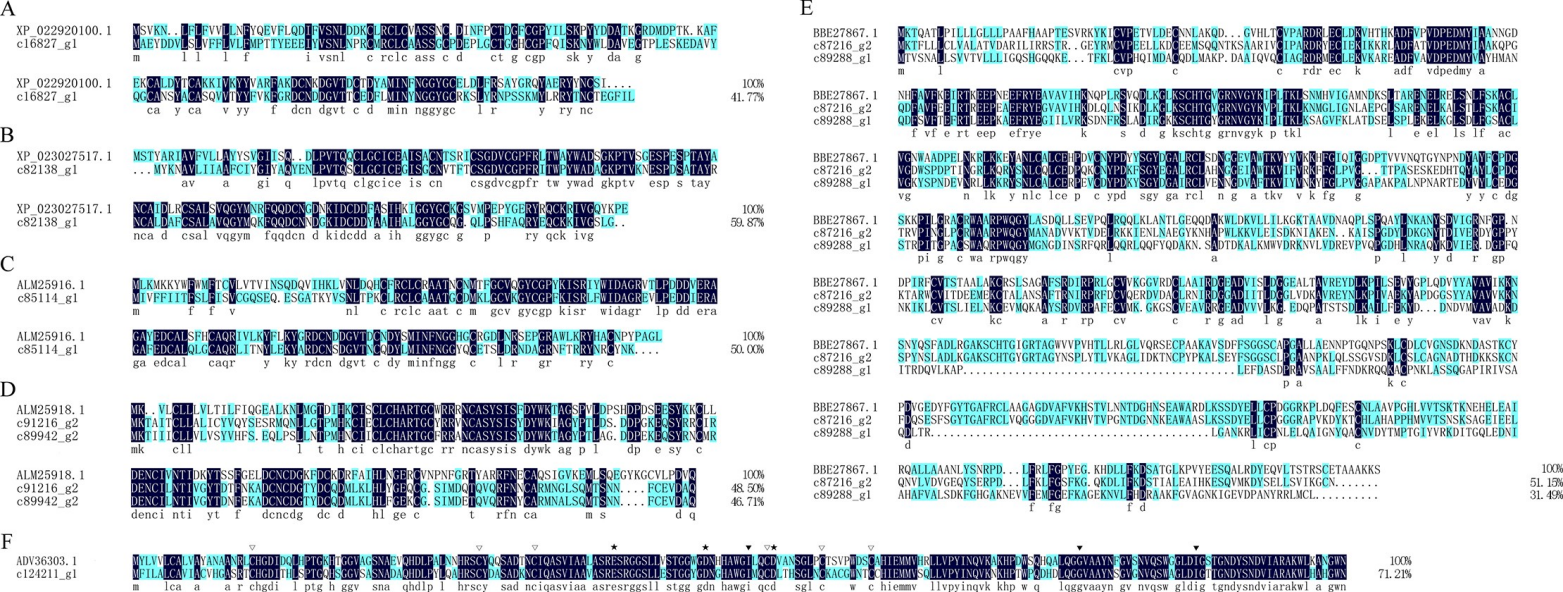
Alignment of the amino acid sequences of putative lysozymes from *Protaetia brevitarsis Lewis* Larvae with known sequences. XP_022920100.1: i-type lysozyme from *Onthophagus Taurus*. XP_023027517.1: i-type lysozyme from *Leptinotarsa decemlineata*. ALM25916.1 and ALM25918.1: i-type lysozyme from *Harmonia axyridis*. BBE27867.1: c-type lysozyme from *Locusta migratoria*. ADV36303.1: g-type lysozyme from *Physella acuta*; Catalytic residues and the substrate binding sites of g-type lysozymes are marked with black pentagon (★) and black triangle (▼), respectively; and white triangles (▽) show the six conserved cysteine residues.

Lysozymes are a group of enzymes with antibacterial activity that ubiquitously exist in diverse organisms, including invertebrates and vertebrates. They can catalyze the hydrolysis of β-1,4-glycosidic bonds between *N*-acetylglucosamine and *N*-acetylmuramic acid in the peptidoglycan of bacterial cell walls, which causes bacterial cell lysis and thereby prevents bacterial infections [36]. Based on structural and functional features, lysozymes are categorized into three major groups in the animal kingdom: invertebrate-type (i-type) lysozymes, chicken-type (c-type) lysozymes, and goose-type (g-type) lysozymes [37]. The c- and g-type lysozymes are found in various vertebrate and invertebrate species [38]. And the g-type lysozymes have a transglycosylase SLT domain (Bacterial lytic transglycosylases Domain) which contains three conserved catalytic binding sites, three conserved substrate binding sites, and six conserved cysteine residues. In this study, five of these unigenes (c16827_g1, c82138_g1, c85114_g1, c89942_g2, c91216_g2) were annotated as i-type lysozymes, which is similar to the number found in the beetles *Harmonia axyridis* and *Meligethes aeneus* [39]. As shown in Fig 5, the unigene c16827_g1 shares 41.77% identity with the i-type lysozyme (XP_022920100.1) from the beetle *Onthophagus Taurus* (Fig 5A), c82138_g1 shares 59.87% identity with the i-type lysozyme (XP_023027517.1) from the beetle *Leptinotarsa decemlineata* (Fig 5B), c85114_g1 shares 50.00% identity with the i-type lysozyme (ALM25916.1) from the beetle *Harmonia axyridis* (Fig 5C), and c89942_g2 and c912216_g2 share 46.71% and 48.50% identity with the i-type lysozyme (ALM25918.1) from the beetle *Harmonia axyridis* (Fig 5D). Two of these unigenes (c87216_g2, c89288_g1) were annotated as c-type lysozymes, and they shared 51.15% and 31.49% identity with a c-type lysozyme (BBE27867.1) from *Locusta migratoria* (Fig 5E). One of these unigenes (c124211_g1) was annotated as a g-type lysozyme, and it shares 71.21% identity with a g-type lysozyme (ADV36303.1) from *Physella acuta* (Fig 5F), which has shown activity against both *E*. *coli* and MRSA [40].

#### Defensin

**Fig 6:**
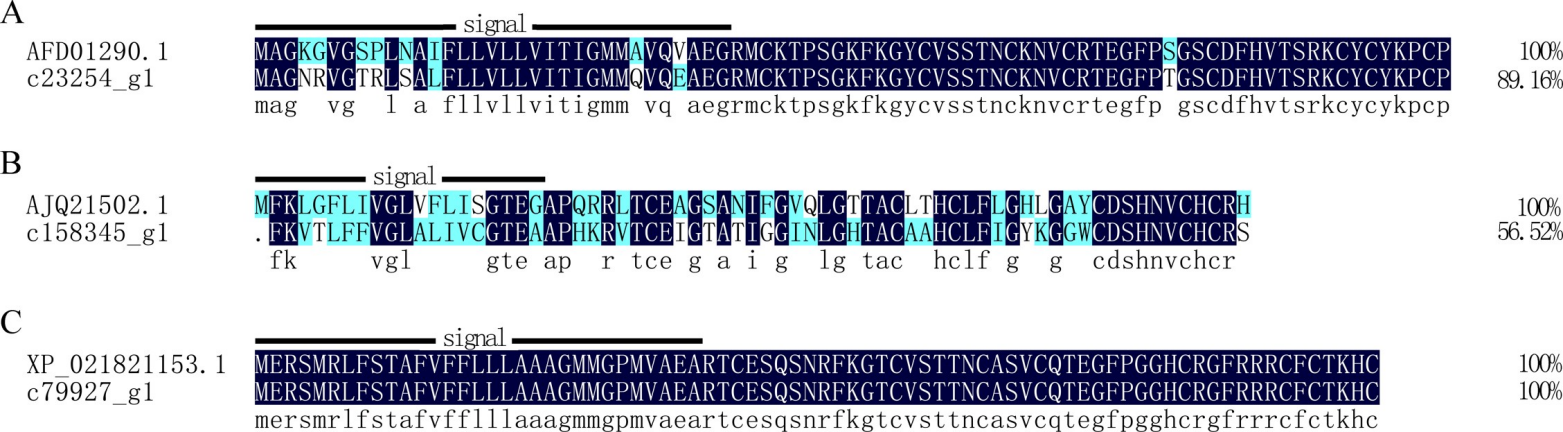
Alignment of the amino acid sequences of putative defensin and defensin-like proteins from *Protaetia brevitarsis Lewis* Larvae with known sequences. AFD01290.1: defensin-3 from *Pinus sylvestris*. AJQ21502.1: defensin-like 1 protein from *Mytilus galloprovincialis*; XP_021821153.1: defensin-like protein from *Prunus avium*.

Defensins are small, cationic, and cysteine-rich peptides that include 3–4 intramolecular disulfide bonds. Hundreds of insect defensins have been identified since the first defensins were reported in the flesh fly *Sarcophaga peregrina* [41] and the black blowfly *Phormia terraenovae* [42]. They are mainly active against Gram-positive bacteria, and some are also active against Gram-negative bacteria as well as fungi [43]. In this study, one of these unigenes (c23254_g1) was annotated as a defensin, and two (c158345_g1, c79927_g1) were annotated as defensin-like proteins. As shown in Fig 6, the unigene c23254_g1 shares 89.16% identity with defensin-3 (AFD01290.1) from *Pinus sylvestris* (Fig 6A), c158345_g1 shares 56.52% identity with defensin-like 1 protein (AJQ21502.1) from *Mytilus galloprovincialis* (Fig 6B), and the c79927_g1 sequence was identical to a defensin-like protein (XP_021821153.1) from *Prunus avium* (Fig 6C).

#### PBSIP

**Fig 7:**
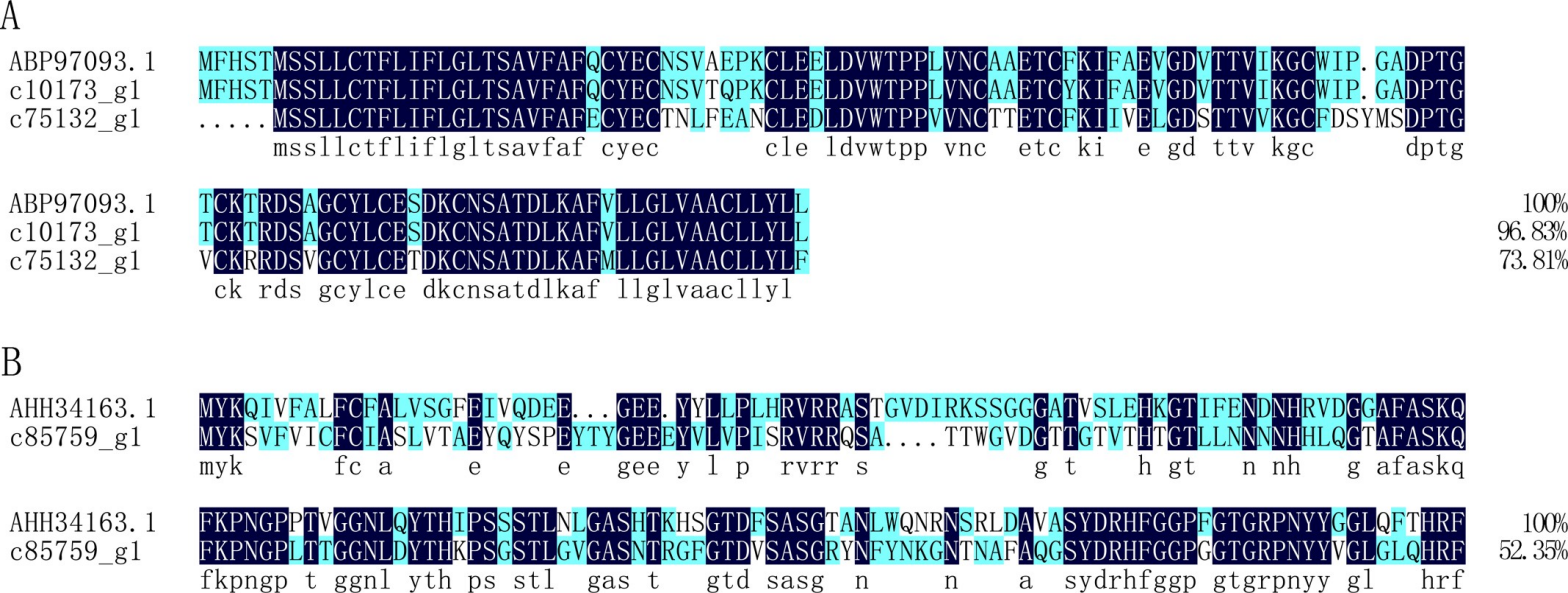
Alignment of the amino acid sequences of putative PBSIP protein and Attacin from *Protaetia brevitarsis Lewis* Larvae with known sequences. A: putative PBSIP protein; ABP97093.1 is the PBSIP from *Protaetia brevitarsis seulensis*. B: putative attacin; AHH34163.1: attacin 2 from *Microdera dzhungarica*.

The antibacterial peptide PBSIP was previously reported in *Protaetia brevitarsis seulensis* [44]. In this study, two unigenes (c10173_g1, c75132_g1) were annotated as matching the antibacterial peptide PBSIP, sharing 96.83% and 73.81% sequence identity with PBSIP (ABP97093.1) from *Protaetia brevitarsis seulensis* (Fig 7A), respectively.

#### Attacin

Attacins are glycine-rich immune proteins originally isolated from the moth *Hyalophora cecropia*, and they have a broad antibacterial range, particularly for Gram-negative bacteria [43]. In this study, one unigene (c85759_g1) was annotated as attacin, and it has 52.35% identity with attacin 2 (AHH34163.1) from *Microdera dzhungarica* (Fig 7B).

#### Coprisin

**Fig 8:**
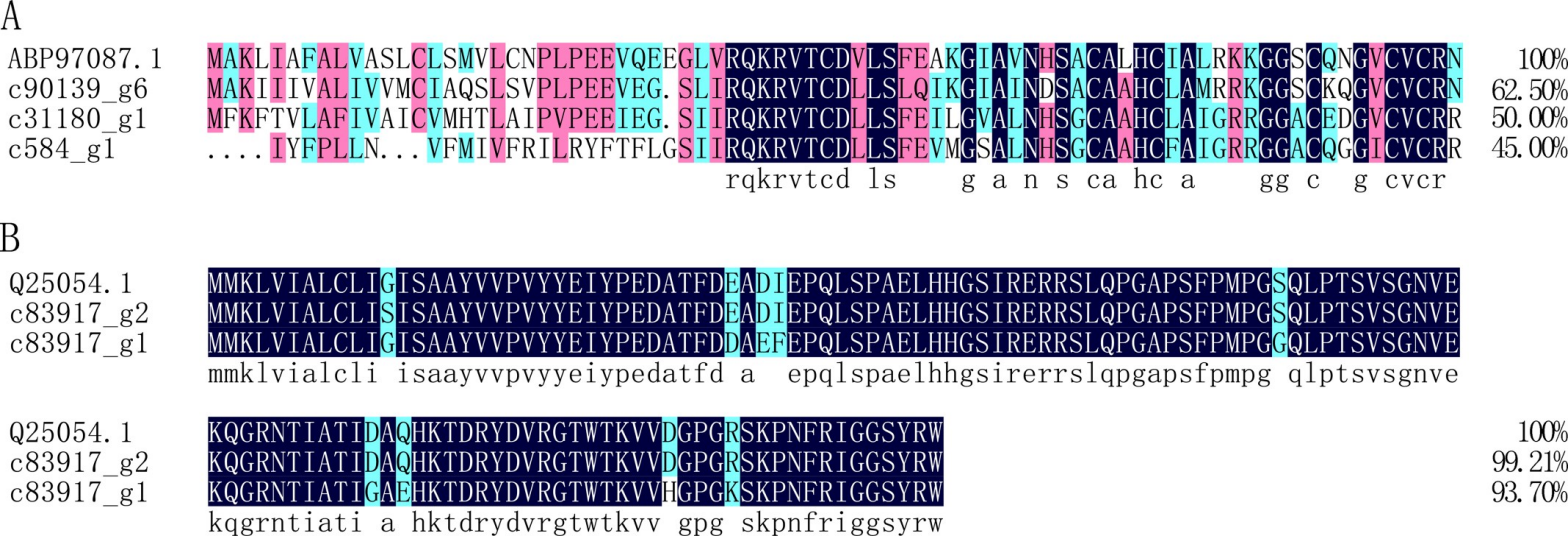
Alignment of the amino acid sequences of putative coprisin and coleoptericin-like protein from *Protaetia brevitarsis Lewis* Larvae with known sequences. A: putative coprisin; ABP97087.1: coprisin from Copris tripartitus. B: putative coleoptericin-like protein; Q25054.1: Holotricin-2 from *Holotrichia diomphalia*.

Coprisin is a cysteine-rich peptide isolated from the dung beetle *Copris tripartitus*, and it has exhibited broad-spectrum activity against both Gram-positive and Gram-negative bacteria [45]. In this study, three unigenes (c31180_g1, c541_g1, c90139_g6) were annotated as coprisin, with 50.00%, 45.00%, and 62.50% identity shared with coprisin (ABP97087.1) from *Copris tripartitus* (Fig 8A), respectively.

#### Coleoptericin-like protein

Coleoptericin-like proteins, including coleoptericin, acaloleptin, holotricin, and rhinocerosin, are glycine- and proline-rich AMPs with bactericidal activity against Gram-positive and Gram-negative bacteria. In this study, two unigenes (c83917_g1, c83917_g2) were annotated as holotricin, and they share 93.70% and 99.21% identity with Holotricin-2 (Q25054.1) from *Holotrichia diomphalia* (Fig 8B), respectively. Additionally, one fragment of a coleoptericin gene (c65461_g1) was similar to coleoptericin A (BAB40436.1) from *Trypoxylus dichotomus*.

#### Thaumatin-like proteins

**Fig 9:**
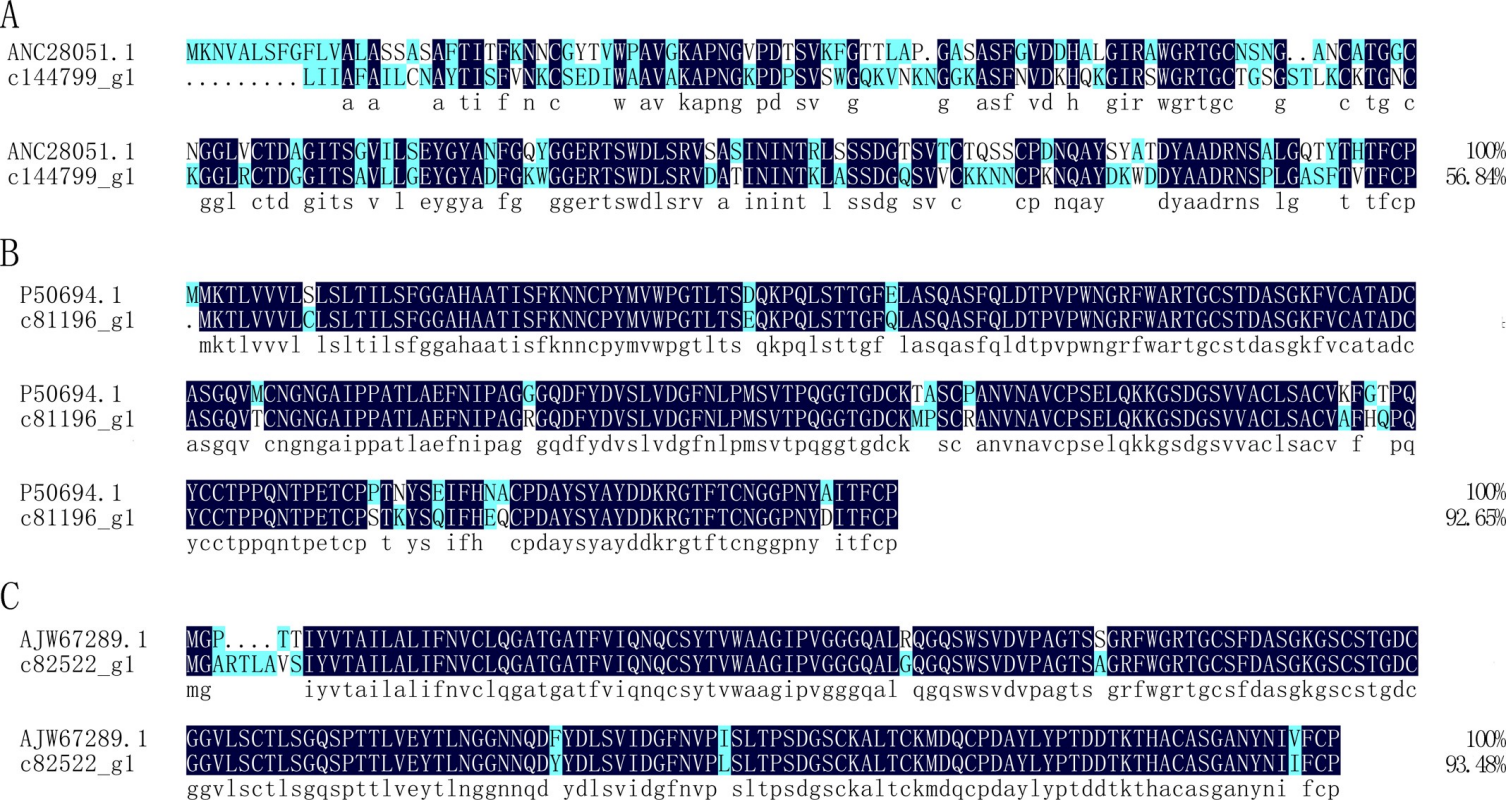
Alignment of the amino acid sequences of putative Thaumatin-like protein from *Protaetia brevitarsis Lewis* Larvae with known sequences. ANC28051.1: thaumatin-like protein from *Polyporus umbellatus*; P50694.1: thaumatin-like protein from *Prunus avium*; AJW67289.1: thaumatin-like protein from *Pinus massoniana*.

Thaumatin-like proteins (TLPs) are polypeptides that share sequence similarity with thaumatin, a sweet-tasting protein originally identified from the fruit of the West African rain forest shrub *Thaumatococcus daniellii* [46]. TLPs are widely distributed in organisms, including fungi, animals, and plants [47]. They exhibit antifungal activity via membrane permeabilization [48], β-glucan binding and degradation [49], inhibition of enzymes [50], and apoptosis [51]. In this study, three unigenes (c114799_g1, c81196_g1, c82522_g1) were annotated as thaumatin-like protein. The unigene c114799_g1 shares 56.84% identity with a thaumatin-like protein (ANC28051.1) from *Polyporus umbellatus* (Fig 9A), c81196_g1 shares 92.65% identity with a thaumatin-like protein (P50694.1) from *Prunus avium* (Fig 9B), and c82522_g1 shares 93.48% identity with a thaumatin-like protein (AJW67289.1) from *Pinus massoniana* (Fig 9C). Additionally, two fragments of peritrophin-like genes (c119696_g1 and c94535_g1) were also identified, with c119696_g1 and c94535_g1 being identical to a thaumatin-like protein (PQQ05260.1) from *Prunus yedoensis* var. *nudiflora* and a thaumatin-like protein (ABE01396.1) from *Camellia sinensis*, respectively.

#### Peritrophin

**Fig 10:**
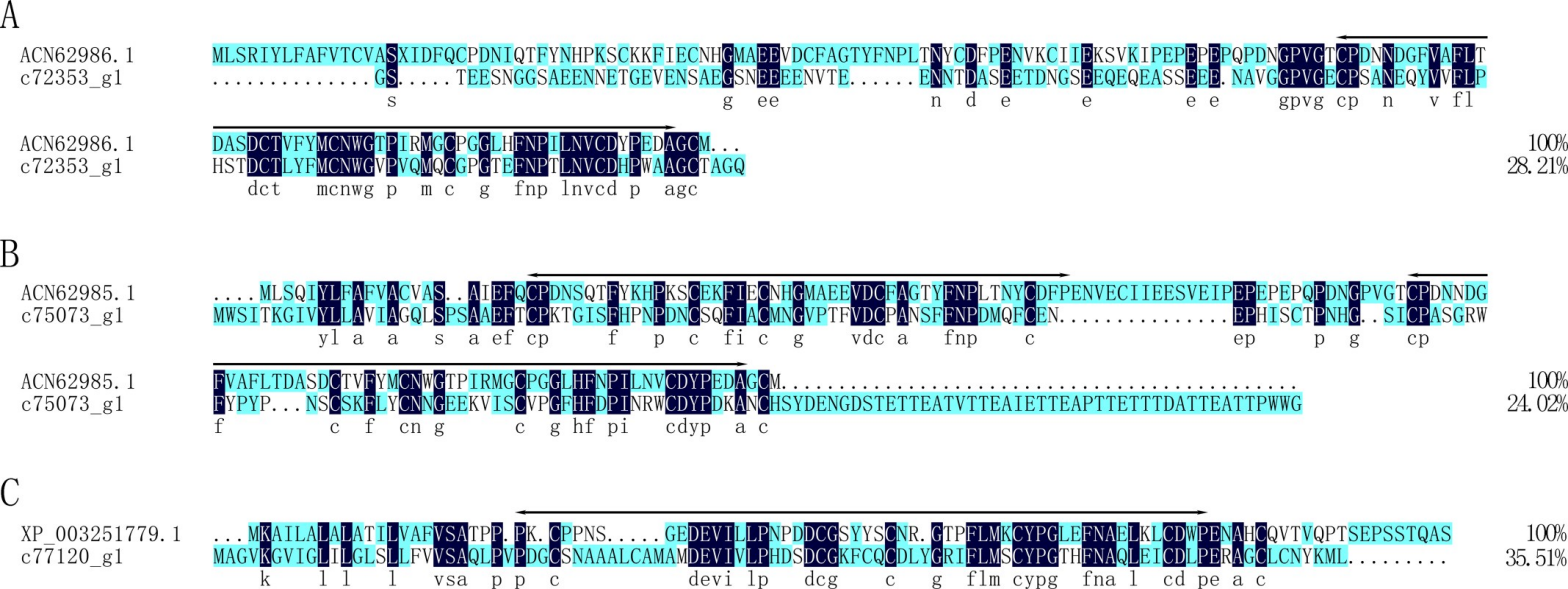
Alignment of the amino acid sequences of putative peritrophin from *Protaetia brevitarsis Lewis* Larvae with known sequences. ACN62986.1: mucin-like peritrophin from *Popillia japonica*; ACN62985.1: peritrophin from *Popillia japonica*; XP_003251779.1: peritrophin-1 protein from *Apis mellifera*. The chitin-binding domains were lined.

Peritrophin was first isolated from insect intestines, where it was inferred to protect insects from microorganismal infection [52]. These proteins have a signal sequence and one of three kinds of peritrophin domains: peritrophin-A domains, peritrophin-B domains, or peritrophin-C domains. Peritrophin-A domains are also referred to as chitin-binding type 2 domains [53]. Today, more and more peritrophin-like proteins have also been found in many organisms [54]. In this study, the unigene c72353_g1 was annotated as a mucin-like peritrophin and shared an identity of 28.21% with a mucin-like peritrophin (ACN62986.1) from *Popillia japonica* (Fig 10A). The unigene c75073_g1 was annotated as peritrophin and shared an identity of 24.02% with peritrophin (ACN62985.1) from *Popillia japonica* (Fig 10B). The unigene c77120_g1 was annotated as peritrophin-1-like protein and shared an identity of 35.51% with the peritrophin-1 (XP_003251779.1) protein from *Apis mellifera* (Fig 10C).

**Fig 11:**
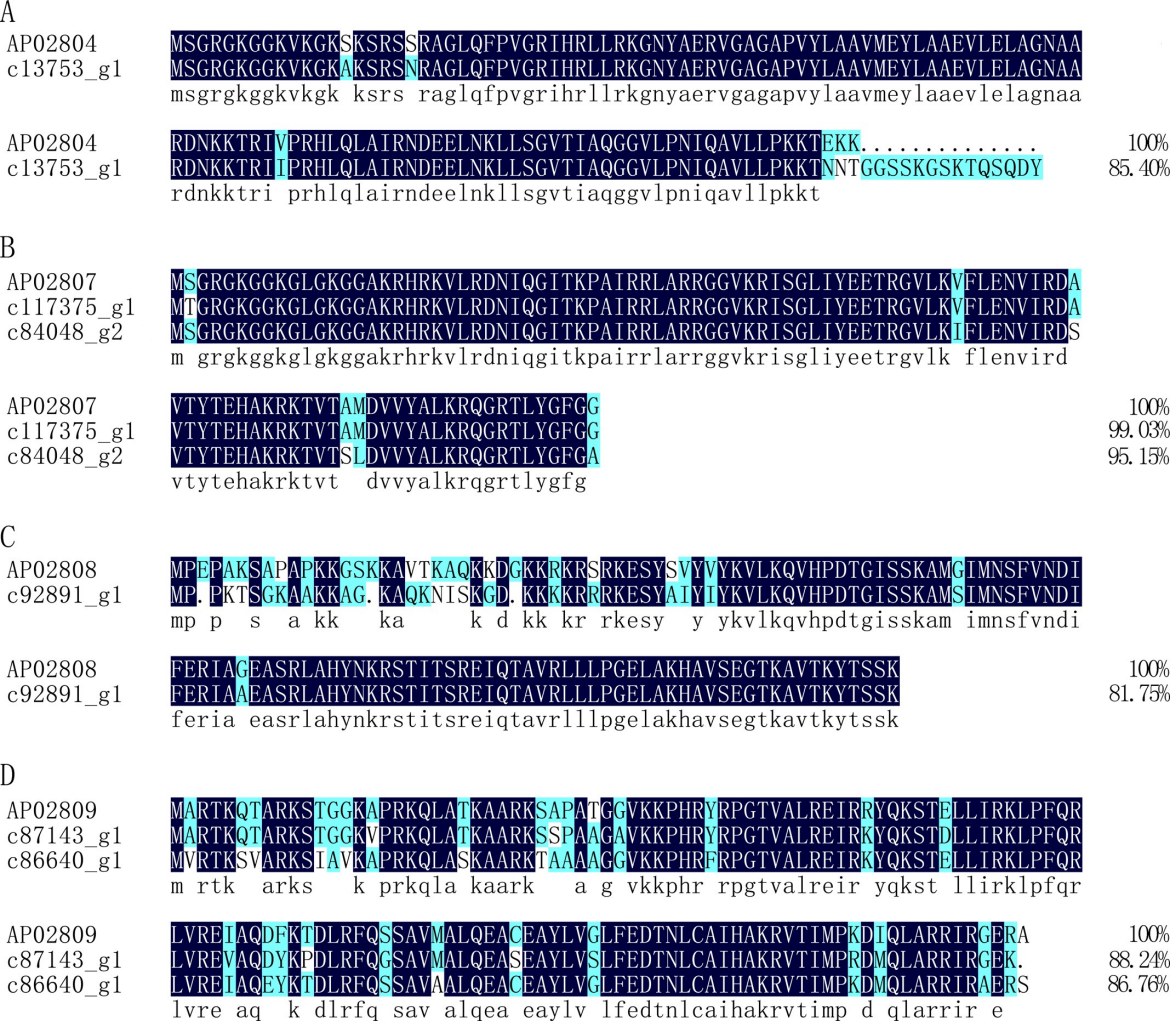
Alignment of the amino acid sequences of putative Histones from *Protaetia brevitarsis Lewis* Larvae with known sequences with antimicrobial activity. AP02804: histone H2A from Pacific white shrimp *Litopenaeus vannamei* in the database APD; AP02807: histone H4 from American cupped oysters *Crassostrea virginica* in the database APD; AP02808: histone H2B from calf thymus *Bos taurus* in the database APD; AP02809: histone H3 from calf thymus *Bos taurus* in the database APD.

Many other proteins and peptide sequence derived from other protein or peptide have also shown antimicrobial activity [55, 56]. To identify more proteins and peptide sequences with potential antimicrobial activity, the transcripts were BLASTed against AMP databases (APD, CAMP, and LAMP) with an identity cut off 80%. Finally, we identified four unigenes that were annotated as histones and shared high identity with histones with antimicrobial activity in the database. As shown in Fig 11, the unigene c13753_g1 shares an identity of 85.40% with the H2A histone (ID in APD: AP02804) from the Pacific white shrimp *Litopenaeus vannamei* (Fig 11A), which has anti-Gram positive bacteria activity [57]. The unigenes c117375_g1 and c84048_g2 share identities of 99.03% and 95.15% respectively, with the H4 histone (ID in APD: AP02807) from American cupped oysters *Crassostrea virginica* (Fig 11B), which has anti-Gram negative bacteria activity [58]. The unigene c92891_g1 shares an identity of 81.75% with the H2B histone (ID in APD: AP02808) from cattle *Bos taurus* (Fig 11C), which has anti-Gram negative bacteria activity [59], while c86640_g1 and c87143_g1 share identities of 86.76% and 88.24%, respectively, with the H3 histone (ID in APD: AP02809) from cattle *Bos taurus* (Fig 11D), which has also shown anti-Gram negative bacteria activity [59]. Furthermore, we also identified 41 peptide sequences (Table E in S1 File) predicted to have potential antimicrobial activity on the basis of sequence similarity.

## Conclusion

This study is the first whole transcriptome analysis of larvae from the non-model species *P*. *brevitarsis*, which accordingly will contribute to the exploration of the species through the resulting genetic resources and will thus facilitate further comprehensive studies of this species. Moreover, the natural AMPs, proteins with potential antimicrobial activity, and potential antimicrobial peptide sequences identified from transcripts in *P*. *brevitarsis* larvae provide templates for further development of new antimicrobial agents and will promote the utilization of the species for its full medical potential.

## Supporting information

S1 File Detailed description of the methods and supplementary tables. (DOCX)

S2 File The final assembly unigenes of *Protaetia brevitarsis Lewis* Larvae. (FASTA)
