# Supplementary material for: The transcriptome analysis of Protaetia brevitarsis Lewis larvae
Source: PLoS One. 2019 Mar 21;14(3):e0214001. doi: 10.1371/journal.pone.0214001 (PMC6428405; doi:10.1371/journal.pone.0214001)
Supplement: S1 File — (DOCX) [file pone.0214001.s001.docx]

**The Transcriptome Analysis of *Protaetia brevitarsis Lewis* Larvae**

Zhongjie Li**^*^**, Miaomiao Meng, Shasha Li, Bo Deng

*Medical College, Henan University of Science and Technology, Luoyang 471023, PR China*

**^*^** Corresponding authors. E-mail: sui_feng5217@126.com

**Methods**

**cDNA library construction and Illumina sequencing**

Total RNA was extracted using TRIzol reagent (Life Technologies, Carlsbad, CA, USA), and RNA degradation and contamination was monitored on 1% agarose gels. The NanoPhotometer® spectrophotometer (Implen, CA, USA) was used to check RNA purity. RNA concentration was measured using Qubit® RNA Assay Kit in Qubit® 2.0 Fluorometer (Life Technologies, CA, USA). And RNA integrity was assessed using the RNA Nano 6000 Assay Kit of the Bioanalyzer 2100 system (Agilent Technologies, CA, USA).

RNA samples from the six individuals were pooled in equal amounts to generate a mixed sample for library construction. The sequencing library was generated using NEBNext® Ultra™ RNA Library Prep Kit for Illumina® (NEB, USA) following manufacturer’s recommendations and index codes were added to attribute sequences to each sample. In order to select cDNA fragments of preferential 150~200 bp in length, the library fragments were purified with AMPure XP system (Beckman Coulter, Beverly, USA) and library quality was assessed on the Agilent Bioanalyzer 2100 system.

The clustering of the index-coded samples was performed on a cBot Cluster Generation System using TruSeq PE Cluster Kit v3-cBot-HS (Illumia) according to the manufacturer’s instructions. After cluster generation, the library preparations were sequenced on an Illumina HiSeq TM2000/ MiSeqTM platform and paired-end reads were generated.

**De novo assemble and gene annotation**

At first, raw data (raw reads) of Fastq format were processed through in-house Perl scripts to remove reads containing adapter, reads containing ploy-N and reads of low quality. And Q20, Q30, GC-content and sequence duplication level of the clean data were calculated. All the downstream analyses were based on clean data with high quality.

Transcriptome de novo assembly was carried out using the program Trinity (Grabherr, Haas et al. 2011) with min_kmer_cov set to 2 by default and all other parameters set default. And gene annotation was based on the databases including NCBI Nr (non-redundant protein sequences), Nt (non-redundant nucleotide sequences), Pfam (Protein family), KOG (euKaryotic Ortholog Groups of proteins), Swiss-Prot (A manually annotated and reviewed protein sequence database), KO (KEGG Ortholog database) and GO (Gene Ontology). To obtain significant annotations, alignments with Nr, Nt and Swiss-Prot databases were carried out with a cut-off E-value of 10^-5^, KEGG classification was done with a cut-off E-value of 10^-10^, KOG classification was done with a cut-off E-value of 10^-3^, and GO classification was done with a cut-off E-value of 10^-6^.

**Table A. Statistics of Illumina sequencing in the transcriptome of *Protaetia brevitarsis Lewis* Larvae.**

| Statistics project | Number |
| --- | --- |
| Raw reads | 52,991,910 |
| Clean reads | 50,796,336 |
| Clean bases (Gb) | 7.62 |
| Error (%) | 0.01 |
| Q20 (%) | 98.09 |
| Q30 (%) | 95.54 |
| GC (%) | 42.88 |

**Table B. Statistics of de novo assemble for the transcriptome of *Protaetia brevitarsis Lewis* Larvae.**

|  | Items | |
| --- | --- | --- |
|  | Transcripts | Unigenes |
| Total number | 169,087 | 142,000 |
| Total nucleotides | 100,655,359 | 68,499,259 |
| 200-500bp number | 127,056 | 114,932 |
| 500-1,000bp number | 20,106 | 14,974 |
| 1,000-2,000bp number | 12,298 | 7,452 |
| >2,000bp number | 9,627 | 4,642 |
| Mean length | 595 | 482 |
| Min length | 201 | 201 |
| Median length | 289 | 274 |
| Max length | 27,601 | 27,601 |
| N50 | 1023 | 561 |
| N90 | 243 | 233 |

**Table C. The number of unigenes annotated in public database searched.**

|  | Number of unigenes | Percentage (%) |
| --- | --- | --- |
| Annotated in Nr | 36,743 | 25.87 |
| Annotated in Nt | 12,881 | 9.07 |
| Annotated in KO | 16,368 | 11.52 |
| Annotated in SwissProt | 28,727 | 20.23 |
| Annotated in PFAM | 40,511 | 28.52 |
| Annotated in GO | 41,921 | 29.52 |
| Annotated in KOG | 21,454 | 15.1 |
| Annotated in at least one Database | 56,937 | 40.09 |
| Annotated in all Databases | 4,618 | 3.25 |

**Table D. Frequency of SSRs based on motif types in *Protaetia brevitarsis Lewis* Larvae**

| SSR motif type | Number of repeats | | | | | | | | | Total | Percentage |
| --- | --- | --- | --- | --- | --- | --- | --- | --- | --- | --- | --- |
|  | 5 | 6 | 7 | 8 | 9 | 10 | 11 | 12 | ≥13 |  |  |
| A/T | - | - | - | - | - | 6232 | 2706 | 1479 | 2778 | 13195 | 68.92 |
| C/G | - | - | - | - | - | 106 | 58 | 25 | 166 | 355 | 1.85 |
| AC/GT | - | 180 | 48 | 34 | 15 | 7 | 10 | 2 | - | 296 | 1.55 |
| AG/CT | - | 199 | 82 | 53 | 26 | 19 | 23 | 3 | - | 405 | 2.12 |
| AT/AT | - | 448 | 210 | 104 | 48 | 33 | 14 | 4 | - | 861 | 4.50 |
| CG/CG | - | 11 | 2 | - | - | - | - | - | - | 13 | 0.07 |
| AAC/GTT | 97 | 36 | 18 | 4 | - | - | - | 1 | - | 156 | 0.81 |
| AAG/CTT | 410 | 179 | 76 | 4 | - | - | - | - | - | 669 | 3.49 |
| AAT/ATT | 679 | 376 | 148 | 4 | - | - | - | - | - | 1207 | 6.30 |
| ACC/GGT | 169 | 58 | 27 | 4 | - | - | - | - | 1 | 259 | 1.35 |
| ACG/CGT | 84 | 32 | 10 | 4 | - | - | - | - | - | 130 | 0.68 |
| ACT/AGT | 38 | 8 | 8 | 3 | - | - | - | - | - | 57 | 0.30 |
| AGC/CTG | 100 | 34 | 23 | 3 | 1 | - | - | - | - | 161 | 0.84 |
| AGG/CCT | 645 | 254 | 98 | 4 | - | - | - | - | - | 1001 | 5.23 |
| ATC/ATG | 78 | 35 | 16 | 5 | - | - | - | - | - | 134 | 0.70 |
| CCG/CGG | 78 | 26 | 5 | 5 | - | - | - | - | - | 114 | 0.60 |
| AAAC/GTTT | 6 | 1 | - | - | - | - | - | - | - | 7 | 0.04 |
| AAAG/CTTT | 6 | - | - | - | - | - | - | - | - | 6 | 0.03 |
| AAAT/ATTT | 32 | 2 | - | - | - | - | - | - | - | 34 | 0.18 |
| AACC/GGTT | 2 | 1 | - | - | - | - | - | - | - | 3 | 0.02 |
| AACT/AGTT | 1 | - | - | - | - | - | - | - | - | 1 | 0.01 |
| AAGG/CCTT | 2 | 2 | - | - | - | - | - | - | - | 4 | 0.02 |
| AAGT/ACTT | 1 | - | 1 | - | - | - | - | - | - | 2 | 0.01 |
| AATC/ATTG | - | 1 | - | - | - | - | - | - | - | 1 | 0.01 |
| AATG/ATTC | 16 | 3 | - | - | - | - | - | - | - | 19 | 0.10 |
| AATT/AATT | 4 | - | - | - | - | - | - | - | - | 4 | 0.02 |
| ACAG/CTGT | - | 1 | - | - | - | - | - | - | - | 1 | 0.01 |
| ACAT/ATGT | 8 | 2 | - | - | - | - | - | - | - | 10 | 0.05 |
| ACTC/AGTG | 1 | - | - | - | - | - | - | - | - | 1 | 0.01 |
| ACTG/AGTC | 2 | - | - | - | - | - | - | - | - | 2 | 0.01 |
| AGAT/ATCT | 6 | 3 | - | - | - | - | - | - | - | 9 | 0.05 |
| AGCC/CTGG | - | 1 | - | - | - | - | - | - | - | 1 | 0.01 |
| AGGC/CCTG | 1 | - | - | - | - | - | - | - | - | 1 | 0.01 |
| AGGG/CCCT | - | 1 | - | - | - | - | - | - | - | 1 | 0.01 |
| ATCC/ATGG | 2 | - | - | - | - | - | - | - | - | 2 | 0.01 |
| ATCG/ATCG | 1 | - | - | - | - | - | - | - | - | 1 | 0.01 |
| AAAAT/ATTTT | 2 | - | - | - | - | - | - | - | - | 2 | 0.01 |
| AAACT/AGTTT | 1 | - | - | - | - | - | - | - | - | 1 | 0.01 |
| AAAGG/CCTTT | - | - | - | - | - | - | - | - | 1 | 1 | 0.01 |
| AAAGT/ACTTT | - | - | 1 | - | - | - | - | - | - | 1 | 0.01 |
| AAATG/ATTTC | 1 | - | - | - | - | - | - | - | - | 1 | 0.01 |
| AAGCC/CTTGG | - | - | - | - | - | 1 | - | - | - | 1 | 0.01 |
| AATCT/AGATT | 1 | - | - | - | - | - | - | - | - | 1 | 0.01 |
| ACACC/GGTGT | - | 1 | - | - | - | - | - | - | - | 1 | 0.01 |
| ACCAG/CTGGT | 1 | - | - | - | - | - | - | - | - | 1 | 0.01 |
| AGATG/ATCTC | 1 | - | - | - | - | - | - | - | - | 1 | 0.01 |
| AGGCG/CCTCG | 1 | - | - | - | - | - | - | - | - | 1 | 0.01 |
| AAAACC/GGTTTT | - | 1 | - | - | - | - | - | - | - | 1 | 0.01 |
| AAAGAG/CTCTTT | - | 1 | - | - | - | - | - | - | - | 1 | 0.01 |
| AAGAGG/CCTCTT | - | - | - | - | - | - | - | - | 1 | 1 | 0.01 |
| AATGGG/ATTCCC | - | - | - | 1 | - | - | - | - | - | 1 | 0.01 |
| ACCACG/CGTGGT | - | - | - | - | - | - | - | - | 1 | 1 | 0.01 |
| ACGATG/ATCGTC | 1 | - | - | - | - | - | - | - | - | 1 | 0.01 |
| AGCGGG/CCCGCT | - | 1 | - | - | - | - | - | - | - | 1 | 0.01 |
| AGGGGC/CCCCTG | - | - | - | - | - | 1 | - | - | - | 1 | 0.01 |
| CCCCCG/CGGGGG | - | - | 1 | - | - | - | - | - | - | 1 | 0.01 |

**Table E. Predicted antimicrobial peptide sequence.**

| Unigene ID | Sequence | AMPs database/ID | AMPs sequence |
| --- | --- | --- | --- |
| c130918_g1 | **GKDSGKAKAKAVSRSQRAGLQFPVGRIHRHLK**NRTTSHGRVGATAAVYSAAILEYLTAEVLELAGNASKDLKVKRI | APD/AP02814 | MAGGKAGKDSGKAKAKAVSRSARAGLQFPVGRIHRHLK |
| c70810_g1 | TFNEM**ARTKQTARKSTGGKAPRKQLPS**ALLLPLPFNSRCQGCPQVCPHRRHWWCQEAPPLPSRYRRSP | APD/AP02791 | ARTKQTARKSTGGKAPRKQLAT |
| c83433_g1 | **TKQTARKSTGGKAPRKQLAT**KAARKSAPTTGGVKKPHRYRPGTVALREIRKYQKSTELLIRKLPFQRLVREIAQDFKTDLRFQSHAVLALQEAAEAYLVGLFEDTNLCAIHAKRVTIMPKDIQLARRIRGERA | APD/AP02791 | ARTKQTARKSTGGKAPRKQLAT |
| c87143_g1 | IQM**ARTKQTARKSTGGKVPRKQLAT**KAARKSSPAAGAVKKPHRYRPGTVALREIRKYQKSTDLLIRKLPFQRLVREVAQDYKPDLRFQGSAVMALQEASEAYLVSLFEDTNLCAIHAKRVTIMPRDMQLARRIRGEK | APD/AP02791 | ARTKQTARKSTGGKAPRKQLAT |
| c101576_g1 | CFDRVKMGFVMGCAVGMAAGALFGTF**SCLRIGMRGRELMGGIGKT**MMQSGGTFGTFMAIGMGIRC | CAMP/CAMPSQ7581 | SCLRIGMRGRELMGGVGKT |
| c101827_g1 | ESKVDQFGRKSGFWCSFLQNLRGFELRM**PNPKVFFDMQVGGAPAGRIVMELYA**DVVPKTAENFRALCTGEKGTGRSGKPLHFKGSSFHRVIPGFMCQ | CAMP/CAMPSQ3985 | PNPKVFFDMTIGGQSAGRIVMEEYA |
| c102357_g1 | QKAGLQFPVGRIARFLKAGKYAERVGAGAPVYLAAVLEYLAAE**VLELAGNAARDNKKNRIVPRHIQL**AVRNDEELSKLLGAVTIANGGVLPNIHQVLLPKKSGKDKGEIGSASQEF | CAMP/CAMPSQ8148 | ILELAGNAARDNKKTRIIPRHLQL |
| c120585_g1 | AVYTAAVLEYLAAE**VLELAGNAARDNKKTRIIPRHIQL**AVRNDDELGKLLGKVTIASGGVLPNIHQVLLPKKVAEKSEKSAAPKS | CAMP/CAMPSQ8148 | ILELAGNAARDNKKTRIIPRHLQL |
| c129815_g1 | NIRLSKFFSFVAACGTEAIKMQLHIRGQATHVLECQGTELIGQIKEQIASLEQLQASEVSLYAAGCPVSDDCLVSEFGSTDLEISVGLLGG**KVHGSLARAGKVKGQTPKVDKQEKKKKKTGRAKRRIQYNRRFVNVVTTFGRRRGPNSNS**A | CAMP/CAMPSQ3754 | KVHGSLARAGKVRGQTPKVAKQEKKKKKTGRAKRRMQYNRRFVNVVPTFGKKKGPNANS |
| c13753_g1 | AKVIVNPVLSYLLNM**SGRGKGGKVKGKAKSRSNRAGLQFPVGRIHRLLRKGNY**AERVGAGAPVYLAAVMEYLAAEVLELAGNAARDNKKTRIIPRHLQLAIRNDEELNKLLSGVTIAQGGVLPNIQAVLLPKKTNNTGGSSKGSKTQSQDY | CAMP/CAMPSQ277 | AGRGKQGGKVRAKAKTRSSRAGLQFPVGRVHRLLRKGNY |
| c13753_g1 | AKVIVNPVLSYLLNMSGRGKGGKVKGKAK**SRSNRAGLQFPVGRIHRLLRK**GNYAERVGAGAPVYLAAVMEYLAAEVLELAGNAARDNKKTRIIPRHLQLAIRNDEELNKLLSGVTIAQGGVLPNIQAVLLPKKTNNTGGSSKGSKTQSQDY | CAMP/CAMPSQ278 | TRSSRAGLQFPVGRVHRLLRK |
| c137829_g1 | **MQIFVKTLTGKTITLEVESSDTIDNVKAKIQDKEGIPPDQQRLIFAGKQLEDGRTLADYNIQKESTLHLVLRLR**GGMQIFV | CAMP/CAMPSQ3702 | MQIFVKTLTGKTITLEVEPSDTIENVKAKIQDKEGIPPDQQRLIFAGKQLEDGRTLSDYNIQKESTLHLVLRLR |
| c23330_g1 | KKTAAKTAVDGERAKPK**TRSSRAGLQFPVGRLHRFLRE**GRYGERIGGGAPVFMAAVLEYLTVEVLELAGNAARDNKKTRISPRHIMLAVRNDEELNQLLSNITIA | CAMP/CAMPSQ278 | TRSSRAGLQFPVGRVHRLLRK |
| c23330_g1 | KKTAAKTAVDGERAKPKTRSSRAGLQFPVGRLHRFLREGRYGERIGGGAPVFMAAVLEYLTVE**VLELAGNAARDNKKTRISPRHIML**AVRNDEELNQLLSNITIA | CAMP/CAMPSQ8148 | ILELAGNAARDNKKTRIIPRHLQL |
| c31818_g1 | EKAKPKTDPTKKRERSKKASKPQSTKSRSYKAGLQFPVGRIHRMLRNGNYADRIGAGAPIYLASVLEYLTAE**ILELAGNASRDNKKQRIIPRHILL**AIRNDEELNKLLKNVTVSAGGVIPNIHTVLLPKKSSQRAAAEEAGSQEY | CAMP/CAMPSQ8148 | ILELAGNAARDNKKTRIIPRHLQL |
| c46460_g1 | YLYIVM**SGRGKGGKVKGKAKSRSSRAGLQFPVGRIHRLLRKGNY**AERVGAGAPVYLAAVMEYLAAEVLELAGNAARDNKKTRIIPRHLQLAIRNDEE | CAMP/CAMPSQ277 | AGRGKQGGKVRAKAKTRSSRAGLQFPVGRVHRLLRKGNY |
| c46460_g1 | YLYIVMSGRGKGGKVKGKAK**SRSSRAGLQFPVGRIHRLLRK**GNYAERVGAGAPVYLAAVMEYLAAEVLELAGNAARDNKKTRIIPRHLQLAIRNDEE | CAMP/CAMPSQ278 | TRSSRAGLQFPVGRVHRLLRK |
| c46460_g1 | YLYIVMSGRGKGGKVKGKAKSRSSRAGLQFPVGRIHRLLRKGNYAERVGAGAPVYLAAVMEYLAAE**VLELAGNAARDNKKTRIIPRHLQL**AIRNDEE | CAMP/CAMPSQ8148 | ILELAGNAARDNKKTRIIPRHLQL |
| c46460_g1 | YLYIVM**SGRGKGGKVKGKAKSRSSRAGLQFPVGRIHRLLRKGNYAERVGAGAPVYL**AAVMEYLAAEVLELAGNAARDNKKTRIIPRHLQLAIRNDEE | CAMP/CAMPSQ853 | SGRGKTGGKARAKAKTRSSRAGLQFPVGRVHRLLRKGNYAHRVGAGAPVYL |
| c65449_g1 | RGG**MQIFVKTLTGKTITLEVEPSDTIENVKAKIQDKEGIPPDQQRLIFAGKQLEDGRTLADYNIQKESTLHLVLRLR**GGIIEPSLMALARKYNQDKMICRKCYARLHPRAVNCRKKKCGHSNQLRPKKKV | CAMP/CAMPSQ3702 | MQIFVKTLTGKTITLEVEPSDTIENVKAKIQDKEGIPPDQQRLIFAGKQLEDGRTLSDYNIQKESTLHLVLRLR |
| c69940_g1 | NCCITMS**KVHGSLARAGKVRGQTPKVAKQDKKKKPRGRAHKRMQYNRRFVTAVVTFGKKRGPNSSE**K | CAMP/CAMPSQ3754 | KVHGSLARAGKVRGQTPKVAKQEKKKKKTGRAKRRMQYNRRFVNVVPTFGKKKGPNANS |
| c80597_g1 | EISALFQSIPKGPTMQPLTVICFLALCTGAITSAYPQEPVLADEARPFANSLFDELPEETYQAAVENFRLKRATCDLLSGFGVGDS**ACAAHCIARGNRGGYCNSKK**VCVCRN | CAMP/CAMPSQ79 | ACAAHCLLRGNRGGYCNGKG |
| c82956_g1 | ASSLSFYPNLFANNPNKPSKFASMA**SNPKVFFDMTIGGQPGGRIVMELYA**DTTPRTAENFRALCTGEKGVGRSGKPLHYKGSSFHRVIPGFMCQGGDFTAGNGTGGESIYGAKFADENFVKKHTGPGILSMANAGPGTNGSQFFVCTAKTEWLDGKHVVFGQVTEGLDVVKNIEKVGSSSGRTSKPVVVADCGQLS | CAMP/CAMPSQ3985 | PNPKVFFDMTIGGQSAGRIVMEEYA |
| c88222_g2 | TLHLVLRLRGG**MQIFVKTLTGKTITLEVENADTIESVKQKIQDKEGIPPDQQRLIFAGKQLEDGRTLQDYNIQKEATLHLVLRL** | CAMP/CAMPSQ3702 | MQIFVKTLTGKTITLEVEPSDTIENVKAKIQDKEGIPPDQQRLIFAGKQLEDGRTLSDYNIQKESTLHLVLRLR |
| c89130_g1 | SKPMAPKAKKPETASPKPRVEVAKKTPKVKTGDKKTK**SRSERAGLQFPVGRIHRLLSK**GNYAERIGAGAPVYLAAVLEYLTAEVLELAGNASRDHKKHRIIPRHIQLAIRNDEELNKFLAHITVPSGGVLPNIHGYLIPKKSKTGEEAEA | CAMP/CAMPSQ278 | TRSSRAGLQFPVGRVHRLLRK |
| c89245_g1 | ESVKQKIQDKEGIPPDQQRLIFAGKQLEDGRTLQDYNIQKEATLHLVLRLRGG**MQIFVKTLTGKTITLEVENADTIESVKQKIQDKEGIPPDQQRLIFAGKQLEDGRTLQDYNIQKEATLHLVLRLR**GG**MQIFVKTLTGKTITLEVENADTIESVKQKIQDKEGIPPDQQRLIFAGKQLEDGRTLQDYNIQKEATLHLVLRLR**GGVIEPTLAALARKYNCEKMVCRKCYARLPARAHNCRKRKCGHSSYIRPKKKGGGK | CAMP/CAMPSQ3702 | MQIFVKTLTGKTITLEVEPSDTIENVKAKIQDKEGIPPDQQRLIFAGKQLEDGRTLSDYNIQKESTLHLVLRLR |
| c89245_g2 | TLQDYSIQKDSTLHLVLRLRGG**MQIFVKTLTGKTITLEVESSDTIDQVKQKIQDKEGIPLDQQRLIFAGKQLEDGRTLSDYNIQKESTLHLVLRL** | CAMP/CAMPSQ3702 | MQIFVKTLTGKTITLEVEPSDTIENVKAKIQDKEGIPPDQQRLIFAGKQLEDGRTLSDYNIQKESTLHLVLRLR |
| c105641_g1 | TMQLFVVAGASHLAEFGASPTVADVKRYVAGVEGLLEADQVVCHCGVPLSDDAGLLSCGVGPMSTLSVTSRLLGG**KVHGSLARAGKVRSQTPK**KDKQEKSKRVTGRAKRRAQYNK | LAMP/L01A001072 | KVHGSLARAGKVRGQTPK |
| c131269_g1 | QNFLSAPSAVIFLFCKSCVSITM**GHQNIWYSHPRKYGQGSRSCRSCSNRHGLIRKYGLNICRQCFREYAHDIGFKKLD** | LAMP/L01A001986 | GHQQLYWSHPRKFGQGSRSCRVCSNRHGLIRKYGLNMCRQCFRQYAKDIGFIKLD |
| c148504_g1 | RFTRKFI**MQNDAGEFVDLLIPRKCSASNRIISAKDHASIQINIAEVDETTGRMTGASKTYALCGAIRRMGESDDSLIRLAKRDGIIAKNF** | LAMP/L01A002337 | MQNDAGEFVDLYVPRKCSASNRIIGAKDHASIQMNVAEVDKVTGRFNGQFKTYAICGAIRRMGESDDSILRLAKADGIVSKNF |
| c39424_g1 | ETMG**KVHGSLARAGKVRGQTPKVAKQDKKKKPRGRAHKRMQYNRRFVTAVVGFGKKRGPNSSEK** | LAMP/L01A001987 | KVHGSLARAGKVRGRHQKVAKQDKKKKPRGRAHKRLQHNRRFVTAVVGFGKKRGPNSSEK |
| c54236_g1 | LLSLAPQRFLRLCFWFLRSTTTK**MVLQNDIDLLHPPAELEKRKHKLKRLVQSPNSFFMDVKCQGCFNITTVFSHSQTVVVCGNCQTVLCQPTGGRARLTEGCSFRRKGD** | LAMP/L01A002345 | MVLSSDIDLLNPPAELEKTKHKRKRLVQSPNSFFMDVKCQGCFNITTVFSHSQTVVMCGSCSSVLCTPTGWPRRLTEGCSFRRKSD |
| c66469_g1 | LQSQPLDNQR**MDSTIKHAVVVKVMGRTGSRGQVTQVRVKFLDDQNRFIMRNVKGPVREGDILTLLESEREARRLR** | LAMP/L01A001983 | MDTQVKLAVVVKVMGRTGSRGQVTQVRVKFLDDQNRLIMRNVKGPVCEGDILTLLESEREARRLR |
| c69940_g1 | NCCITMS**KVHGSLARAGKVRGQTPKVAKQDKKKKPRGRAHKRMQYNRRFVTAVVTFGKKRGPNSSEK** | LAMP/L01A001987 | KVHGSLARAGKVRGRHQKVAKQDKKKKPRGRAHKRLQHNRRFVTAVVGFGKKRGPNSSEK |
| c73229_g1 | SVAMAMAKFFAALILALLAISMLQTTVMANHGNGGHHGGNNAYGPGSL**KSYQCPSQCSRRCGQTQYHKPCMFFCQKCCKKCLCVPPGYYGNKAVCPCYNNWKTQQGGPKCP** | LAMP/L01A000878 | KSYQCGGQCTRRCSNTKYHKPCMFFCQKCCAKCLCVPPGTYGNKQVCPCYNNWKTQQGGPKCP |
| c73229_g1 | SVAMAMAKFFAALILALLAISMLQTTVMANHGNGGHHGGNNAYGP**GSLKSYQCPSQCSRRCGQTQYHKPCMFFCQKCCKKCLCVPPGYYGNKAVCPCYNNWKTQQGGPKCP** | LAMP/L06AT00236 | GSLKSYQCPSQCSRRCSKTQYHKPCMFFCQKCCKKCLCVPPGYYGNKAVCPCYNNWKTKEGGPKCP |
| c80597_g1 | EISALFQSIPKGPTMQPLTVICFLALCTGAITSAYPQEPVLADEARPFANSLF**DELPEETYQAAVENFRLKRATCDLLSGFGVGDSACAAHCIARGNRGGYCNSKKVCVC**RN | LAMP/L03A000008 | DELPEETYQAAVENYRRKRATCDLLSGFGVGDSACAAHCIARRNRGGYCNAKTVCVC |
| c80597_g1 | EISALFQSIPKGPT**MQPLTVICFLALCTGAITSAYPQEPVLADEARPFANSLFDELPEETYQAAVENFRLKRATCDLLSGFGVGDSACAAHCIARGNRGGYCNSKKVCVCRN** | LAMP/L03A000168 | MKSITVICFLALCTVAITSAYPQEPVLADEARPFANSLFDELPEETYQAAVENFRLKRATCDLLSGFGVGDSACAAHCIARGNRGGYCNSKKVCVCRN |
| c80597_g1 | EISALFQSIPKGPTMQPLTVICFLALCTGAITSAYPQEPVLADEARPFANSLFDELPEETYQAAVENFRLKR**ATCDLLSGFGVGDSACAAHCIARGNRGGYCNSKKVCVCR**N | LAMP/L05ADEF414 | ATCDLLSGMGVNHSACAAHCVLRGNRGGYCNSKAVCVCR |
| c81181_g1 | KKTMHIFVKTLTEQTFAFEAEASLSVAEVNMMIESAQGIPCDQQALILNGKRLSEEDVIEESCTLNLTCNLLGGG**KVHGSLSRAGKVRGQTPK**VEKAEKTGKDARGRAKKRKQYTRRFVNVVVTPGARKVGPNNFAARMAREAAAAAKKE | LAMP/L01A001072 | KVHGSLARAGKVRGQTPK |
| c91100_g2 | KRPSFFSDSCCARNAPKVSKMQLHIRGLNTHVLDVQPQESISQIKARLAALENVEDAAHLVLSCEGAILATDALVTDLSSIELDLTIPLLGG**KVHGSLARAGKVKGQTPK**VEKKEKKKKTNNKKILAQTGMKVTIKHLLLKERGYGKVG | LAMP/L01A001072 | KVHGSLARAGKVRGQTPK |
